# Comparison of the inhibitory effects of three transcriptional variants of *CDKN2A *in human lung cancer cell line A549

**DOI:** 10.1186/1756-9966-29-74

**Published:** 2010-06-17

**Authors:** Wei Zhang, Jing Zhu, Jing Bai, Hui Jiang, Fangli Liu, An Liu, Peng Liu, Guohua Ji, Rongwei Guan, Donglin Sun, Wei Ji, Yang Yu, Yan Jin, Xiangning Meng, Songbin Fu

**Affiliations:** 1 Laboratory of Medical Genetics, Harbin Medical University, Harbin 150081, China; 2 Key Laboratory of Medical Genetics (Harbin Medical University), Heilongjiang Higher Education Institutions, Harbin 150081, China

## Abstract

**Background:**

The tumor suppressor gene *CDKN2A *generates at least three different transcriptional variants, each of which is thought to encode a tumor suppressor. However, the inhibitory activities of these variants have not yet been compared in the same cells. Protein therapy is known to have several advantages over gene therapy. Thus, investigation of the exogenous protein molecule of the most effective suppressor may yield meaningful information regarding protein-based cancer therapy.

**Methods:**

The inhibitory effects of *p16INK4a*, *p14ARF *and *p12 *were studied in the human lung cancer cell line A549 which lacks the *CDKN2A *locus. The eukaryotic expression plasmids of the three transcriptional variants were constructed and stably transfected into the cells. RNA and protein expression by the plasmids was confirmed using RT-PCR and fluorescence immunocytochemistry, respectively. Cell growth inhibition and cell-cycle redistribution after transfection were investigated based on growth curve and flow cytometry analyses. An exogenous His-tag fusion p16INK4a protein was obtained and purified by affinity chromatography. Cell growth inhibition and cell cycle arrest induced by the expression of p16INK4a protein were measured in A549 cells transduced with the exogenous protein.

**Results:**

While all three variants suppressed cell growth, *p16INK4a *had the strongest effect. Marked G1-phase accumulation and S-phase inhibition were induced by *p16INK4a *and *p14ARF *but not by *p12*. Exogenous p16INK4a protein was successfully expressed and purified and transduction of the fusion protein into A549 cells inhibited cell growth by G1→S arrest.

**Conclusions:**

Among the three transcript variants, *p16INK4a *has a greater inhibitory effect than *p14ARF *and *p12*; exogenous p16INK4a protein should be further investigated for use in cancer therapy as a protein agent.

## Background

The cell cycle is a strictly ordered process regulated by positive regulators, including cyclins and cyclin-dependent kinase (CDKs), and by negative regulators, such as cyclin-dependent kinase inhibitors (CKIs) [1]. There are two tyepes of CKIs: the INK4 family, which includes *CDKN2A*, and the CIP/KIP family, of which, p21, directly inducible by p53, is an example. Cell cycle regulators are frequently mutated in many types of cancers such that cancer is now considered a cell cycle disease[2]. Accordingly, cell cycle regulators have become an important focus in carcinogenesis research and cancer therapy.

**Figure 1:**
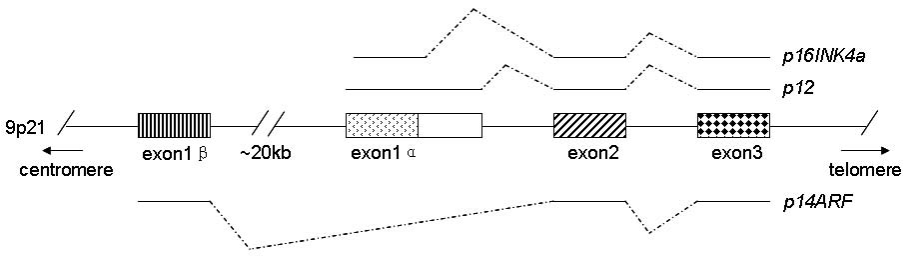
**The three transcriptional variants of *CDKN2A***. The *CDKN2A *gene located at 9p21 generates three transcriptional variants at transcription: *p16INK4a*, *p14ARF *and *p12*. *p16INK4a *utilizes exon1α, and *p14ARF *utilizes exon 1β which is about 20 kb upstream of exon 1α. *p16INK4a *and *p14ARF *share common exon 2 and exon 3 but use different reading frames. *p12 *uses an alternative splice donor site within intron1 of *p16INK4a*.

The tumor suppressor gene *CDKN2A*, located at 9p21, generates at least three structurally and functionally unrelated transcriptional variants: *p16INK4a*, *p14ARF *and *p12 *[3]. In terms of structure, *p16INK4a *and *p14ARF *share the exon 2 and 3 but use unique first exons and utilize different reading frames. *p16INK4a *utilizes exon 1α and *p14ARF *utilizes exon 1β which is 20 kb upstream of exon 1α. *p12 *is a splice variant of an alternative donor splice site within intron 1 of *p16INK4a *which contains exon1α and a novel intron-1-encoded C-terminus[4]. (Figure 1). The protein products of these transcripts function via different pathways. p16INK4a specifically binds to the cyclin-dependent kinases CDK4/6, thereby inhibiting the phosphorylation of the retinoblastoma protein (pRB) and causing cell-cycle arrest at the G1 phase [5]. p14ARF interacts with MDM2, which targets p53 for degradation, thereby inducing p53-dependent cell-cycle arrest in both G1 and G2 phases [6,7]. p53 participates in a wide range of activities including growth arrest, DNA repair and apoptosis and nearly 50% of human tumors have defects in p53 [8]. Less is known about p12; pRB-independent growth suppression by p12 was reported in pancreatic cells, but the tumor suppressive and cell-cycle effects of this protein are as yet unclear [4].

The *CDKN2A *locus is frequently inactivated in a wide variety of tumors[9-12]. Kamb examined 290 tumor cell lines and detected *CDKN2A *deletion in 133 of them [13]. Park examined 31 non-small cell lung cancer (NSCLC) cell lines and found that the inactivation rate of *p16INK4a *and *p14ARF *was 84% and 55% respectively. Significantly, *p16INK4a *was inactivated in all cell lines in which *p14ARF *was inactivated[14]. Conversely, restoration of the transcripts in tumors with endogenous expression deficiency has been shown to reverse the malignant phenotypes of many tumors. In lung cancer cells, for examples, Zhang X et al restored the expression of *p16INK4a *in A549 cells and showed that *p16INK4a *could suppress cell growth and block G1-S cell cycle transition both in vitro and in vivo[15]. Elevated p16INK4a protein expression also enhanced the sensitivity to cisplatin treatment of NSCLC cells[16]. Xie Qi-chao et al co-transfected *p16INK4a *and *p14ARF *into the A549 cells and found that cell growth arrest and apoptosis were induced [17]. As for *p12*, little is known about its status and tumor-suppressive effects. Keith et al transfected a *p12 *eukaryotic expression vector into C-33A and PANC-1 cells and found that the expression of the protein suppressed cell growth by 40% and 60%, respectively, and found no relationship with RB state. While all three transcripts are potential tumor suppressors in different genetic backgrounds, they may have different effects and mechanisms. So far, the activity of the transcriptional variants under the same condition has not been studied, nor is it known which variant has the strongest suppression effect.

Inactivation of the *CDKN2A *locus has been shown to efficiently impair expression of the three transcripts simultaneously [18]. Thus, in tumors deficient in all three transcripts, it is of interest to compare the effects of each one against the same tumor background, considering the potential significance of the findings with respect to cancer therapy. Accordingly, the aim of the present study was to individually restore expression of the three transcripts in a lung-cancer cell line with endogenous expression deficiency and then to compare the inhibitory effects of each one. Distinguishing the different effects of the *CDKN2A *variants will identify whether they differ in their growth-inhibiting effects. This approach will, in addition, reveal the function of *p12 *in lung cancer cells

Along with gene therapy, the use of protein therapeutic agents is rapidly developing[19,20]. More encouragingly, protein therapy has been shown to overcome the drawbacks of vector-associated toxicity and immune responses associated with gene therapy and to avoid its delayed therapeutic impacts due to the need for transcription and translation of the encoded protective protein[21]. It is therefore meaningful to identify the most effective and useful suppressor for future applications as a protein therapeutic agent.

Here, the different growth inhibition effects of *p16INK4a*, *p14ARF *and *p12 *were investigated in a study that included the exogenous expression, purification and function of the p16INK4a protein. Our results demonstrated the different effects of the three transcripts on cell growth and their activity at different phases of the cell cycle. Among the three variants, *p16INK4a *was shown to more effectively suppress the growth of A549 lung cancer cells. Our research on the p16INK4a protein could facilitate or improve the basic understanding of future cancer biotherapy with the p16INK4a protein.

## Methods

### Cell culture

The human lung cancer cell line A549, deficient in the *CDKN2A *locus and wild-type in *RB *and *p53 *[22], was obtained from the Cell Resource Center of the Shanghai Academy of Sciences The cells were cultured in F12-K medium (Sigma-Aldrich, St.Louis, MO) supplemented with 10% fetal bovine serum (FBS) (GIBCO BRL) in a humidified 5% CO_2 _air incubator at 37°C.

### Plasmids construction and stable transfection

Full-length fragments of complementary DNA (cDNA) corresponding to *p16INK4a*, *p14ARF *and *p12 *were obtained by reverse transcription polymerase chain reaction (RT-PCR) from AGZY and H446 cells and normal pancreas tissue, respectively, which were positive for the respective transcript. The PCR products were cloned into pGEM-T vector (Promega, Medison, WI). The PCR products were cloned into the vector pGEM-T (Promega, Medison, WI) and the transcripts PCR-amplified using primers containing the same restriction-enzyme sites as the clone vector plasmids. Primers for *p16INK4a *were 5'-CCCAAGCTTGCATGGAGCCGGCGGCG-3' and 5'-CGGGATCCCTTTCAATCGGGGATGT-3'. Primers for *p14ARF *were 5'-CCCAAGCTTAGATGGGCAGGGGGCGG-3' and 5'-CGGGATCCCTCCTCAGCCAGGTCCA-3'. Primers for *p12 *were 5'-CCCAAGCTTGCATGGAGCCGGCGGCG-3' and 5'-CGGGATCCCCTCATTCCTCTTCCTT-3'. The PCR products and pcDNA3 vector were double-digested with *HindIII *and *BamHI *(TaKaRa, Japan). Eukaryotic expression plasmids were constructed, verified by DNA sequencing, and then used to transfect A549 cells using the Lipofectamine 2000 transfection reagent (Invitrogen, Carlsbad, CA). Transfection of the empty pcDNA3 vector served as the control. The stably transfected cells were screened by adding 600 mg G418/L for 14 days. Positive cell clones were selected and gene expression subsequently confirmed by RT-PCR (with the same primers as described above) and fluorescence immunocytochemistry analyses.

### Protein expression, purification and transduction

*p16INK4a *cDNA was PCR-amplified from clone vector plasmids with primers 5'-TACCGAGCTCGGATCCCGGAGAG-3' and 5'-GTCTCGAGCATGCATCTAGAG-3'. The *p16INK4a *cDNA and the pQE-31 vector (QIAGEN) were double-digested with *BamHI *and *SphI *(TaKaRa, Japan). The PQE31-p16INK4a plasmid was constructed and transformed into BL21(DE3) competent cells. The positive clone (confirmed by DNA sequencing) was grown at 37°C in LB medium supplemented with 100 mg ampicillin/L until the absorbance at 600 nm reached 0.6. Protein expression was induced overnight at 25°C with isopropy-β-D-thiogalactoside (IPTG) at a final concentration of 0.1 mmol/L. The Cells were harvested, resuspended in 20 mL lysis buffer (0.5 M/L NaH_2_PO_4_, 0.5 M/L Na_2_HPO_4_, 29.3 g NaCl/L, pH 7.4), lysed by ultrasonication and centrifuged at 12,000 ×g for 30 minutes at 4°C. The supernatant was loaded onto a Ni^2+^-Agarose column. Nonspecific binding was removed with washing buffer (50 mmol Na_2_HPO_4_/L, 0.3 mol NaCl/L, 10--50 mmol imidazole/L, pH 8.0). The His-tag fusion p16INK4a protein was eluted with elution buffer (50 mmol Na_2_HPO_4_/L, 0.3 mol NaCl/L, 20--200 mmol imidazole/L, pH 8.0). Purified protein was analyzed by 12% SDS-PAGE and Western-blotting. Protein was transduction into A549 cells using Lipofectamine 2000 reagent. After 6 h of incubation, the culture mixture was replaced with fresh medium. The transduction efficiency was verified by fluorescence immunocytochemistry.

### Western blot analysis

Fifty μg protein was separated by 12% SDS-PAGE and transferred to polyvinylidene difluoride membranes (Immobilon-P; Millipore, Bedford, MA). The membranes were blocked, washed, and then incubated with primary p16INK4a antibody (monoclonal mouse anti-human, Santa Cruz, 1:200) for 1 h, followed by a second wash and incubation with secondary antibody (monoclonal goat anti-mouse, 1:2000) for 1 h. Bands were visualized using an enhanced chemiluminescence (ECL) detection kit (Amersham, UK).

### Fluorescence immunocytochemistry

Plasmids- or protein- transduced cells were seeded on cover slips in 6-well plates at a density of 5 × 10^4 ^cells/mL. After 24 h of incubation, cells adhered to cover slips were washed in cold phosphate-buffered saline (PBS), fixed in 4% paraformaldehyde for 15 min, and permeabilized in PBS with 0.1% Triton X-100 for 15--20 min. The fixed cells were then incubated in 1:200 diluted primary antibodies (anti-human *p16INK4a*/*p12 *was from Santa Cruz (SC-468), and anti-human *p14ARF *from Fujian Maixin Corp., China) at 37°C for 2 h, washed, and incubated for 2 h with 1:50 diluted FITC-conjugated secondary antibodies (Beijing Zhongshan Golden Bridge Biotechnology Corp., China). The pcDNA-vector-transfected cells were stained with anti-p16INK4a/p12 and anti-p14ARF antibodies. The nuclei of A549 cells transduced with p16INK4a protein were counterstained using Hoechst stain.

### Cell growth suppression assays

Transduced cells or control cells were seeded onto 24-well plates at an initial density of 1 × 10^4 ^cells/mL and then trypsinized, harvested, and counted at 24-h intervals (plasmid transfection groups) or 12-h intervals (protein transduction groups). The cell number at each time point was determined in three separate wells and experiments were independently repeated at least three times.

### Cell cycle analysis

The redistribution of cells in the cell cycle was analyzed by flow cytometry analysis. After 48 h of cultivation, transduced cells and the control groups were harvested by trypsinization, washed with PBS and fixed in 75% ethanol at 4°C for 24 h. The cycle TEST⁜ PLUS DNA Reagent Kit (BD Biosciences, San Jose, CA) was used for cell sample preparation and DNA staining according to the manufacturer's guidelines. Cell cycle distribution was analyzed by flow cytometry analysis (Bio-Rad, Richmond, CA). All experiments were repeated at least three times.

### Statistical analysis

All values are expressed as means ± SD. Student's *t*-test was used to assess statistical differences. A *p *value < 0.05 was considered significant.

## Results

### Construction and identification of A549 cell clones stably expressing exogenous *p16INK4a*, *p14ARF *and *p12*

Full-length cDNAs were cloned into pcDNA3 vectors designated as pcDNA3-*p16INK4a*, pcDNA3-*p14ARF*, and pcDNA3-*p12*, verified by DNA sequencing (data not shown), and stably transfected into A549 cells. Positive cell clones were identified by G418 screening for 14 days and the expression of exogenous *p16INK4a*, *p14ARF*, or *p12 *examined by RT-PCR and immunocytochemical assays.

**Figure 2:**
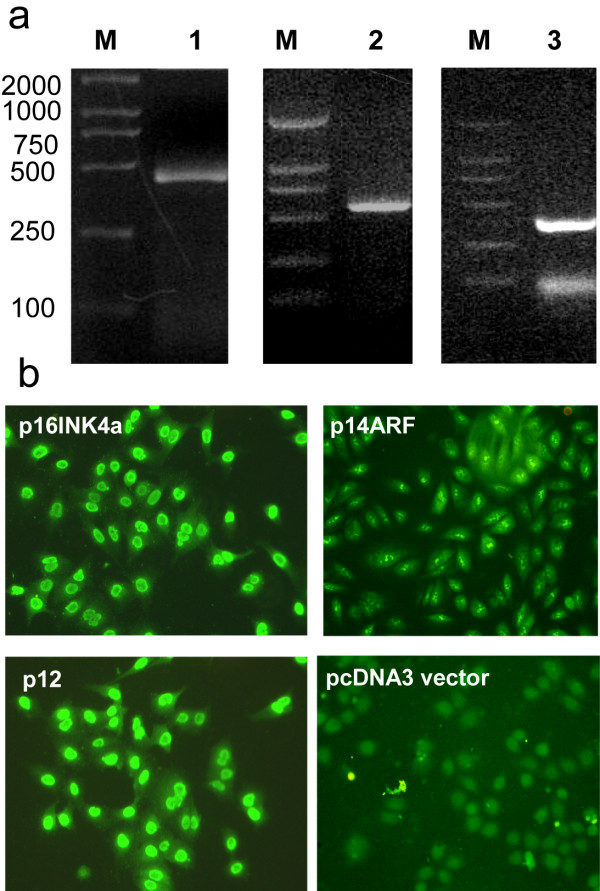
**Identification of stable A549 cell clones for RNA and protein expression**.a. RT-PCR detection of RNA expression of *p16INK4a *(lane 1), *p14ARF *(lane 2) and *p12 *(lane 3). The products were analyzed by 1% agarose gel electrophoresis. Lane M was loaded with DL 2000 DNA marker, with sizes shown on the left. b. Immunocytochemical assays detected expression of *p16INK4a*, *p14ARF *and *p12 *proteins in the cell clones. No specific signal was detected in the negative control (pcDNA3 vector-transfected cells).

RT-PCR of the transfected cells confirmed the presence of products of the expected sizes (493, 543, and 372 bp) (Figure 2a). Immunocytochemical assay results were in agreement with the RT-PCR results and showed significant green fluorescence in cells transfected with each of the three transcripts, thus demonstrating protein expression. The empty-plasmid group stained with anti-p16INK4a/p12 and anti-p14ARF antibodies did not show fluorescence, excluding the background signals (Figure 2b).

### Differential effects of *p16INK4a*, *p14ARF *and *p12 *on growth control of A549 cells

**Figure 3:**
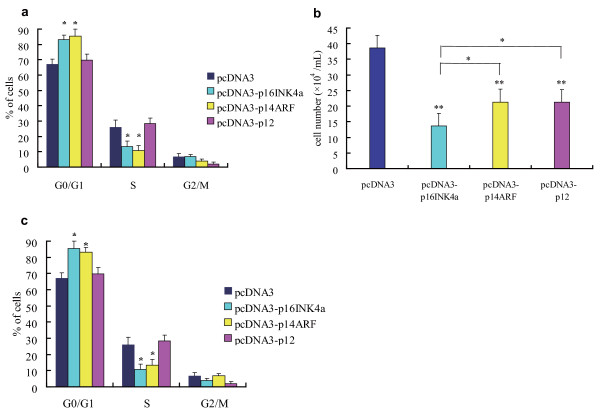
**Cell growth inhibition and cell cycle redistribution analyses of stably transfected A549 cells**. a. Cell growth curve analysis in one representative experiment. Data shown are the mean ± standard deviation of triplicate wells. b. Comparison of cell growth inhibition effects of *p16INK4a*, *p14ARF *and *p12 *on the final day of cell counting, based on three independent experiments. It was shown that all three transcripts significantly suppressed cell growth compared with the empty vector, but *p16INK4a *had the strongest effect. Error bars represent the standard deviation.* *p *< 0.05, ** *p *< 0.01. c. The percentage of stable clone cells at each stage of the cell cycle 48 h after subculture. *p16INK4a *and *p14ARF *induced clear G0/G1-phase accumulation and a decrease in the number of cells in S phase. *p12 *did not have a significant effect on the A549 cell cycle. Data shown are the mean ± standard deviation of three independent experiments. * *p *< 0.05.

Growth arrest effects of the three transcripts were assessed by measuring the growth of the stably transfected clones over a period of 1 week at 24-h intervals. Figure 3a shows a reduction in the growth rate of cells transfected with *p16INK4a*, *p14ARF*, and *p12 *compared with the control group after day 3. During the following 3 days, the growth suppression effects became even more pronounced. As seen in Figure 3b, on the final day of cell counting, proliferation of the cells carrying any one of the three transcriptional variants was significantly inhibited compared to cells carrying the empty expression vector. Moreover, *p16INK4a *had a greater suppressive effect than *p14ARF *and *p12*.

To determine the mechanisms responsible for cell growth suppression, the stable transfected cells were analyzed by flow cytometry, which allowed comparison of the cell cycle distribution of the cells after 48 h of subculture (Figure 3c). Both *p16INK4a *and *p14ARF *induced marked increases in the number of cells in G0/G1 phase and a decrease in the number of those in S phase, whereas pcDNA3-*p12*-transfected cells shows no significant cell cycle changes.

Since *p16INK4a *had the greatest growth suppressive effects, the protein was investigated in further studies, described below.

### Expression of exogenously induced p16INK4a transduced into A549 cells

**Figure 4:**
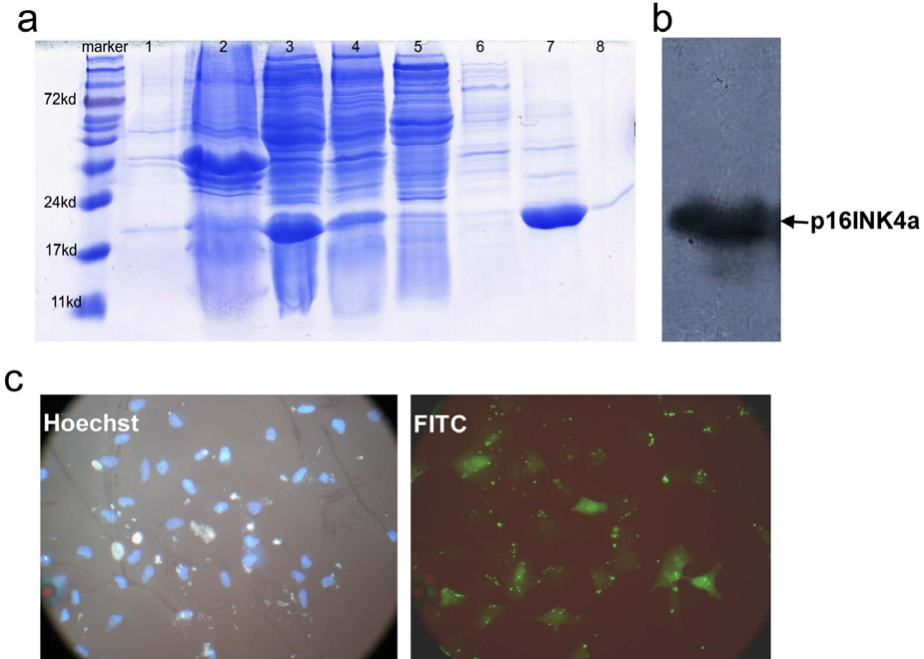
**Purification, verification, and transduction of exogenous p16INK4a fusion protein**. a. Successful expression and purification of the p16INK4a fusion protein was confirmed by 12% SDS-PAGE analysis. The bacterial sample before IPTG induction showed almost no protein expression (lane 1). After IPTG induction and centrifugation, p16INK4a fusion protein was abundant in the clear supernatant (lane 3) (indicated by the arrow) and absent from the bacterial precipitate (lane 2). The supernatant was loaded onto a Ni^2+^-affinity chromatography column, which binds the His-p16INK4a fusion protein. Nonspecifically bound proteins were removed with washing buffer; the flow-through liquid can be seen in lane 4. Elution buffer with different concentrations of imidazole was used to elute the p16INK4a fusion protein: 20 mM (lane 5), 50 mM nt (lane 6), 100 mM (lane 7) and 200 mM (lane 8) were. The fractions were assessed by SDS-PAGE and the sample corresponding to the 100 mM imidazole eluent (lane 7) was found to contain p16INK4a fusion protein of high purity (arrow). b. The purified protein was verified by Western-blot analysis using the specific p16INK4a antibody. c. Immunocytochemical assay to assess transduction efficiency. All nuclei of A549 cells stained with Hoechst fluorescent and the exogenous p16INK4a protein was detected in about 50% of cells, as shown by the FITC signal. As shown in the figure, the transduction efficiency was about 50%.

To produce exogenous p16INK4a protein, plasmid pQE31-p16INK4a-BL21 was generated and confirmed by DNA sequencing. Figure 4a shows the almost complete absence of bacterial protein expression before IPTG induction, whereas after induction, a His-tag fusion protein of approximately 20 kDa was produced that was present in abundance in the supernatant of an extract prepared from the bacterial cells. For protein purification, the supernatant was loaded onto a Ni^2+^-affinity chromatography columns and cell proteins eluted with different concentrations of imidazole. The target protein was found to be enriched in the 100 mM imidazole eluent. All samples were analyzed by 12% SDS-PAGE. The p16INK4a fusion protein was further verified by Western blotting using a specific anti-p16INK4a antibody (Figure 4b).

Purified p16INK4a fusion protein was transduced into A549 cells and transduction efficiency was examined by fluorescence immunocytochemistry. As shown in Figure 4c, all A549 cell nuclei were positive for Hoechst fluorescence and about 50% were positive for FITC, indicating that these cells had been successfully transduced with p16INK4a.

### Growth suppression of A549 cells following p16INK4a induction

**Figure 5:**
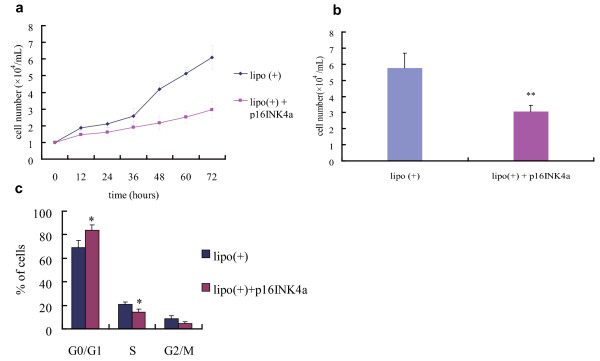
**Cell growth inhibition and cell cycle redistribution effects of p16INK4a in A549 cells**. Cell growth curve in a representative experiment (a) and analysis of the differential inhibition effects of the three variants in three independent experiments carried out after 72 h of subculture (b). Significant growth retardance was exerted by p16INK4a compared with the control. The protein was transduced the day before cell counting. Data shown are the mean ± standard deviation of triplicate wells or experiments. **p < 0.01. c. p16INK4a protein caused evident accumulation of A549 cells in G1 phase and a decrease of those in S phase at 48 h after subculture. Data shown are the mean ± standard deviation of three independent experiments. *p < 0.05.

To evaluate the effect of p16INK4a on cell growth, the growth curves of A549 cells transduced with the protein were compared with those of control cells (A549 cells incubated with Lipofectamine 2000). Cells transduced with p16INK4a the day before the start of the experiment were counted at 12-h intervals. Figure 5a shows that, 36 h after cell subculture, p16INK4a began to induce growth retardation. At 72 h, p16INK4a had significantly suppressed proliferation compared with the control (Figure 5a, b). Furthermore, cell cycle changes, as analyzed by flow cytometry (Figure 5c), showed that the presence of exogenous p16INK4a resulted in a marked retardation of the G1→S transition of A549 cells 48 h after transduction.

## Discussion

As a tumor suppressor, *CDKN2A *is an important gene because its inactivation abrogates two fundamental pathways, that of pRB and p53, both of which are involved in carcinogenesis and tumor progression. So far, the distinct tumor suppressive effects of *p16INK4a *and *p14ARF *have been established, but those of *p12 *have not. Furthermore, to the best of our knowledge, the effects of the three transcripts have not been compared.

The human A549 cell line is a good model to investigate the suppression effects of each of the three transcript variants. The advantages of this cell line are as follows: First, the *CDKN2A *locus is homozygously deleted in this cell line, such that there is no interference from the endogenous proteins. This is an important consideration since the effects of *p16INK4a *were shown in a previous study to be associated with endogenous *p16INK4a *status [23,24]. Previous research in our laboratory also demonstrated that introduction of *p16INK4a *neither suppresses growth nor decreases colony formation rates by Anip973 and AGZY83-a cells expressing endogenous wild-type *p16INK4a *[25]. Second, the A549 cell line is wild-type in *RB *and *p53*. Therefore, *p16INK4a *and *p14ARF *plasmids can be expected to successfully act on the pRB and *p53 *pathways. As to the methods used in this study, the use of stable transfectants confers several advantages as it eliminates a source of variability in transfection efficiency, which facilitated parallel comparison experiments. Furthermore, the characteristics of cells stably transfected with *p16INK4a *have been shown to differ from those transiently transfected with the vector; transient *p16INK4a *transfection induces apoptosis whereas stable transfection markedly suppresses cell growth and cloning efficiency [26].

Research on *p12 *has been hindered as the gene is expressed in normal pancreas tissue, which is difficult to obtain in a well-preserved state. We successfully constructed a eukaryotic expression vector carrying *p12 *and were thus able to show that the gene acts as a proliferation inhibitor in A549 cells. Thus, our research provides evidence that *p12 *has tumor suppressive effects not only in pancreatic and cervical cancer cell lines, as previously reported, but also in a lung cancer cell line. The effects of *p12 *on other cell types will be investigated in future studies. Interestingly, however, *p12 *had no clear effect on the cell cycle of A549 cells. This finding is in accordance with the report that p12 cannot bind cyclin-dependent kinase CDK4 and acts in a pRb-independent manner [4]. The exact mechanism by which *p12 *suppresses cell growth remains to be determined. The *p12 *expression plasmid constructed as part of this study will facilitate investigations into the mechanistic pathway of this transcript.

The different growth suppressive effects of the three transcripts and the possible mechanisms responsible for these differences were compared in growth arrest experiments and by cell cycle analysis. All three transcripts showed significant growth arrest effects compared with the control. Specifically, *p16INK4a *and *p14ARF *caused marked G1-phase accumulation and a decrease in the number of cells in S phase, both of which explain the observed growth inhibition. Notably, *p16INK4a *had the greatest growth suppressive effect among the three variants while the effects of *p14ARF *and *p12 *were similar. This result provides meaningful information in the context of tumor suppressor selection, especially in cells in which *CDKN2A *is inactivated.

As an important complement to gene therapy, protein therapy has its own advantages and its future applications are promising. The administration of protein therapeutic agents has proved to be feasible and effective both in vitro and in vivo [27-29]. In the present study, p16INK4a was exogenously expressed and purified and its tumor suppression effects verified in the A549 cell line. This protein is of interest for the following reasons: First, *p16INK4a *more effectively inhibited cell growth than either *p14ARF *or *p12*. Second, p16INK4a has a low molecular weight, which makes it suitable for protein therapy applications. Third, in contrast to other proteins such as p53, which is involved in a broad range of biological activities, p16INK4a specifically binds CDK4/6. In the present study, the protein was successfully purified and demonstrated to inhibit the proliferation of A549 cells in vitro. The structure and function of p16INK4a will be studied in further investigations, which are likely to provide insight into the use of this protein as a therapeutic agent.

## Conclusions

Our research is the first to show that, although all three transcripts of the *CDKN2A *gene can suppress the growth of lung cancer cells with an inactivated *CDKN2A *locus, they have different effects, with the growth inhibitory effect of *p16INK4a *being the strongest. Inhibitory effects on cell growth by *p16INK4a *and *p14ARF*, but not by *p12*, involve cell cycle redistribution. Thus, p16INK4a may be a candidate agent for cancer biotherapy.

## Competing interests

The authors declare that they have no competing interests.

## Authors' contributions

WZ carried out plasmids construction and stable transfection, cell growth and cell-cycle analyses. FL and AL performed fluorescence immunocytochemistry experiments. HJ, PL and GJ carried out protein expression, purification and transduction experiments. RG and WJ carried out western-blot analyses. JZ wrote the manuscript. XM and DS helped in the modification of the manuscript. JB, YY and YJ participated in the design of the study. All authors read and approved the final manuscript.
